# MRE11 lactylation: a linker between Warburg effect and DNA repair

**DOI:** 10.1093/lifemeta/loae013

**Published:** 2024-04-02

**Authors:** Pingyu Liu, Hongbin Ji, Fuming Li

**Affiliations:** Human Phenome Institute, Zhangjiang Fudan International Innovation Center, Fudan University, Shanghai 201203, China; Institute of Metabolism and Integrative Biology, State Key Laboratory of Cell Biology, Shanghai Institute of Biochemistry and Cell Biology, Center for Excellence in Molecular Cell Science, Chinese Academy of Sciences, Shanghai 200031, China; University of Chinese Academy of Sciences, Beijing 100049, China; School of Life Science, Hangzhou Institute for Advanced Study, University of Chinese Academy of Sciences, Hangzhou, Zhejiang 310024, China; School of Life Science and Technology, Shanghai Tech University, Shanghai 200120, China; Shanghai Key Laboratory of Metabolic Remodeling and Health, Institute of Metabolism and Integrative Biology, Fudan University, Shanghai 200438, China

**Lysine lactylation is a recently discovered post-translational modification with emerging roles in both physiology and disease. A recent study published in *Cell* shows that lactate-induced lactylation of the homologous recombination (HR) protein meiotic recombination 11 results in HR hyperactivation and chemoresistance in cancer cells, establishing a previously unrecognized link between cellular metabolism and deoxyribonucleic acid (DNA) damage repair and providing new insights into how altered glucose metabolism can impact DNA damage response in cancer.**

Cancer cells produce excessive lactate by virtue of aerobic glycolysis, also known as the “Warburg effect” [1]. Once considered as a metabolic waste derived from pyruvate via lactate dehydrogenase (LDH), lactate has now been increasingly appreciated as both a signaling molecule and metabolite fueling cellular metabolism [2]. Importantly, lactate also induces a new post-translational modification (PTM) called lactylation originally identified on histone lysine residues that epigenetically regulates gene transcription [3]. Besides histone lactylation, non-histone lactylation has also progressively been identified, and the physiological and pathological functions of protein lactylation begin to emerge.

**Figure 1:**
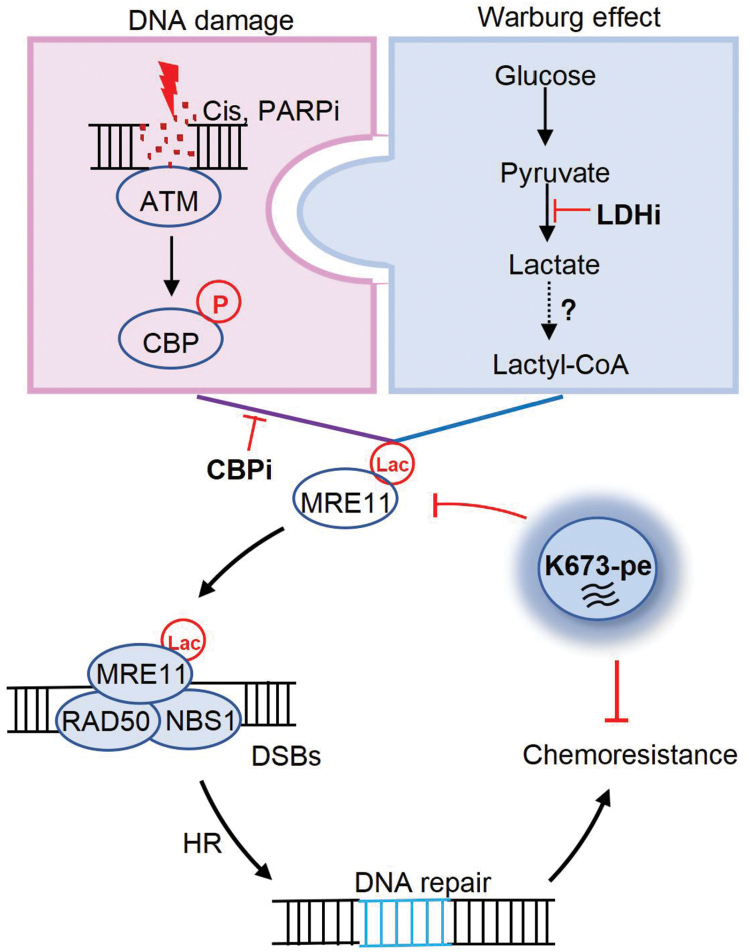
Lactate induces MRE11 lactylation and promotes HR and chemoresistance. In response to DNA damage, ATM phosphorylates CBP to promote MRE11 lactylation using glucose-derived lactate, and MRE11 lactylation facilitates MRN complex binding to DSBs, leading to HR and chemoresistance. CBP or LDH inhibition, and more importantly, K673-pe treatment inhibits MRE11 lactylation, which interferes with HR and results in chemosensitivity.

DNA damage response (DDR) is a complex network of biochemical pathways that cells have evolved to maintain genomic integrity and prevent detrimental mutations from being passed on to their progeny [4]. Deficiencies of DDR cause genomic instability facilitating tumorigenesis and also create liabilities that can be exploited for cancer therapy. While both metabolic deregulation and genomic instability are hallmarks of cancer [5], the interconnection between cellular metabolism and DDR remains largely unknown. For example, it is unclear whether and how the Warburg effect regulates DDR in cancer cells. In a recent study by Chen *et al*., the authors have addressed this question and showed that lactate promotes chemoresistance in cancer by inducing meiotic recombination 11 (MRE11) lactylation to facilitate homologous recombination (HR), one major DNA double-strand break (DSB) repair pathway [6] (Fig. 1).

Intrigued by an initial finding of a negative correlation between lactate-producing enzyme LDH isoform A (LDHA) expression and HR deficiency score in human breast cancer, the authors tested whether protein lactylation directly contributes to HR repair. They found that sodium lactate (NALA) treatment and LDHA pharmacological inhibition positively and negatively affected DNA damage repair, respectively, and functionally altered the sensitivity of cancer cells to chemotherapy. Because NALA treatment increased and LDHA inhibition reduced protein pan-lactylation, these results suggest that protein lactylation potentially regulates DNA damage repair and chemotherapy responses. To identify the HR proteins with lactylation, the authors immunoprecipitated five key HR proteins (MRE11, BRAC1, replication protein A 1 (RPA1), RPA2, and RAD51) followed by immunoblotting with a pan-lactylation antibody. MRE11, the core component of the MRE11/DNA repair protein Rad50 (RAD50)/NBS1 (Nijmegen breakage syndrome 1) (MRN) complex, was found to be lactylated, and MRE11 lactylation levels correlated with lactate availability. Importantly, MRE11 lactylation occurred in an enzyme-dependent manner primarily mediated by the acetyltransferase CREB-binding protein (CBP). Indeed, CBP directly interacted with MRE11, which was promoted by the ataxia-telangiectasia mutated (ATM) kinase-mediated CBP phosphorylation upon DNA damage. The authors further performed mass spectrometry and identified the conserved lysine 673 residue (K673), which is located in the second DNA binding domain of MRE11, as the lactylation site. Using a homemade MRE11 K673 lactylation antibody (MRE11-K673la) with confirmed specificity, they showed that MRE11 K673 lactylation, which took up about 0.5% of total MRE11 levels, was enhanced by NALA treatment and reduced by LDH inhibition, and K673 was the main site of CBP-meditated MRE11 lactylation in response to DNA damage (Fig. 1).

How does lactylation functionally regulate MRE11 during HR? Previous study has shown that MRE11 containing MRN complex senses DNA DSBs and recruits and activates ATM at break sites to orchestrate DNA DDR signaling [7]. Since MRE11 lactylation does not affect the MRN complex formation, the authors tested whether MRE11 lactylation affects its binding to DNA. Results from *in vitro* lactylation assays and electrophoretic mobility shift assays suggested that MRE11 K673 lactylation facilitated MRE11 or MRN complex binding to DNA, whereas ectopic expression of *MRE11 K673R* mutant or pan-lactylation inhibition weakened DNA binding. Using phospho-RPA2 and 5-bromo-20-deoxyuridine (BrdU) foci formation as readouts, the authors further found that DNA end resection was enhanced by NALA treatment and reduced by LDH inhibition, two treatments that correspondingly increased and decreased MRE11 lactylation, respectively. These effects were only seen in MRE11 wild type (WT) but not *K673R* mutant or CBP-depleted cells, supporting that MRE11 lactylation potentially promotes DNA end resection. Notably, MRE11 K673 lactylation does not crosstalk with other PTMs including phosphorylation, ubiquitination, or general control nonderepressible 5 protein (GCN5)-mediated K609 acetylation, suggesting that MRE11 K673 lactylation may directly promote HR. In line with this, *in vitro* data showed that cancer cells expressing *MRE11 K673R* mutant exhibited inefficient DNA damage repair and increased genomic instability compared to those expressing MRE11 WT. Meanwhile, in irradiation-induced tissue damage mouse models, NALA treatment decreased DNA damage while chemical inhibition or genetic deletion of LDHA did the opposite in multiple tissues, collectively supporting that lactatylation regulates DDR both *in vitro* and *in vivo*.

The authors then proceeded to ask whether MRE11 lactylation promotes chemoresistance in cancer cells. They found that NALA treatment made both breast and colon cancer cells resistant to cisplatin or olaparib treatment, whereas CBP or LDH inhibition did the opposite. Moreover, *MRE11 K673R* cells exhibited hypersensitivity to olaparib compared with MRE11 WT cells. These results were further confirmed in cancer patient-derived organoid and xenograft (PDX) models.

Due to the potentially high toxicity of targeting CBP or LDH, the authors sought to develop an alternative strategy specifically targeting MRE11 K673 lactylation for cancer therapy. Based on the sequence around the MRE11 K673 site, they successfully identified one cell penetration peptide (K673-pe) with high efficiency in inhibiting MRE11-K673 lactylation and blunting HR. They found that K673-pe treatment sensitized cancer cells to olaparib and cisplatin treatment *in vitro*. More importantly, K673-pe administration seemed to be well-tolerated *in vivo*, which dramatically increased DNA damage and synergized with olaparib to abrogate tumor growth in a colon cancer PDX model with high MRE11 K673 lactylation. Collectively, these data indicate that targeting MRE11 K673 lactylation with K673-pe could enhance chemotherapy efficacy in cancer with high MRE11 K673 lactylation.

In summary, the authors have unveiled a key role of the “metabolic waste” lactate in regulating HR and chemo-response in cancer cells through protein lactylation. Specifically, lactate induces and phospho-CBP controls MRE K673 lactylation in response to DNA damage, which, in turn, facilitates DNA end resection and HR leading to DNA damage repair and chemotherapy resistance. In this context, inhibition of LDH to reduce lactate availability or CBP inhibition to reduce MRE11 lactylation all dampens HR and increases DNA damage, functionally sensitizing cells to chemotherapy. Importantly, the cell penetration peptide K673-pe directly interferes with MRE11 lactylation and exhibits therapeutic potential by increasing chemotherapy sensitivity (Fig. 1). This study establishes a previously unrecognized link between the Warburg effect and DDR through MRE11 lactylation, and proposes an actionable strategy to enhance chemotherapeutic efficacy by targeting MRE11 lactylation using K673-pe. Considering that the Warburg effect is a general feature of cancer cells, lactate-induced MRE11 lactylation may broadly contribute to DDR and chemoresistance. In this regard, MRE11 K673 lactylation can potentially be used as a biomarker to stratify patients who may benefit from combined chemotherapy and MRE11 lactylation inhibition.

This interesting study also prompts several important questions for future investigation. Firstly, the biochemistry underlying MRE11 lactylation during DNA damage remains to be fully characterized. For example, it remains to be determined whether lactyl-CoA, which is not measured in DNA-damaged cells, is the bona fide metabolic intermediate for MRE11 lactylation, and how lactyl-CoA is generated during DNA damage. Secondly, although MRE11 lactylation promotes DNA binding independent of other PTMs (phosphorylation, ubiquitination, and acetylation) and without affecting MRN complex formation, the underlying mechanisms remain unclear and warrant further investigation. Thirdly, while MRE11 lactylation promotes DNA damage repair and chemoresistance [6], MRE11 has recently been shown to activate cyclic GMP-AMP synthase-stimulator of interferon genes (cGAS-STING)-mediated signaling for tumor suppression through immunosurveillance, which requires MRE11’s DNA binding but not nuclease activity [8]. Given the interconnection between cGAS activation and DNA damage [8], it can be anticipated that MRE11 lactylation might also regulate cGAS activation and immunosurveillance. In this regard, MRE11 may play stage-dependent roles, by limiting tumor initiation through cGAS activation and immunosurveillance and promoting tumor progression and chemoresistance by enhancing DNA damage repair. Accordingly, it is worth evaluating the therapeutic effect of K673-pe in mouse models with intact immune systems. Last but not least, since deregulated metabolism including the Warburg effect is also seen in cellular senescence, it remains possible that MRE11 lactylation regulates cellular senescence based on the strong link of persistent DNA damage to senescence-associated secretory phenotype (SASP) [9]. Strategies that increase MRE11 lactylation may facilitate DNA damage repair and functionally dampen SASP generation, and thus promote healthy aging or prevent aging-associated pathologies.
